# Salivary Defense Proteins: Their Network and Role in Innate and Acquired Oral Immunity

**DOI:** 10.3390/ijms13044295

**Published:** 2012-04-02

**Authors:** Tibor Károly Fábián, Péter Hermann, Anita Beck, Pál Fejérdy, Gábor Fábián

**Affiliations:** 1 Clinic of Prosthetic Dentistry, Faculty of Dentistry, Semmelweis University Budapest, Szentkirályi utca 47, Budapest, H-1088, Hungary; E-Mails: fogpotlastan@fok.usn.hu (P.H.); fejerdy@fok.usn.hu (P.F.); 2 Department of Oral Biology, Faculty of Dentistry, Semmelweis University Budapest, Nagyvárad tér 4, Budapest, H-1089, Hungary; E-Mail: becka@fok.usn.hu; 3 Clinic of Pediatric Dentistry and Orthodontics, Faculty of Dentistry, Semmelweis University Budapest, Szentkirályi utca 47, Budapest, H-1088, Hungary; E-Mail: fabig@fok.usn.hu

**Keywords:** saliva, defense, protein, innate, acquired, immunity, oral

## Abstract

There are numerous defense proteins present in the saliva. Although some of these molecules are present in rather low concentrations, their effects are additive and/or synergistic, resulting in an efficient molecular defense network of the oral cavity. Moreover, local concentrations of these proteins near the mucosal surfaces (mucosal transudate), periodontal sulcus (gingival crevicular fluid) and oral wounds and ulcers (transudate) may be much greater, and in many cases reinforced by immune and/or inflammatory reactions of the oral mucosa. Some defense proteins, like salivary immunoglobulins and salivary chaperokine HSP70/HSPAs (70 kDa heat shock proteins), are involved in both innate and acquired immunity. Cationic peptides and other defense proteins like lysozyme, bactericidal/permeability increasing protein (BPI), BPI-like proteins, PLUNC (palate lung and nasal epithelial clone) proteins, salivary amylase, cystatins, prolin-rich proteins, mucins, peroxidases, statherin and others are primarily responsible for innate immunity. In this paper, this complex system and function of the salivary defense proteins will be reviewed.

## 1. Introduction

### 1.1. Saliva and the Oral Cavity

Saliva is a body fluid, secreted by three pairs of major salivary glands (parotid, submandibular and sublingual) and by many of minor salivary glands [1,2]. Primary saliva is secreted in secretory endpieces (acini) of salivary glands. Primary saliva is modified by serum exudates via tight junctions between several glandular cells (ultrafiltration) and via transcellular diffusion through these cells. Primary saliva is also modified in the intercalated, striated and excretory (collecting) ducts leading from the acini to the mouth. Entering the mouth, ductal saliva of several salivary glands are blended, and supplemented with many constituents that originate from intact or destroyed mucosal cells, immune cells, and oral microorganism [1,2]. Blood constituents also enter the oral cavity via gingival crevicular fluid, via the mucosa as mucosal transudate, and via intraoral bleeding [1,2]. Consequently, a complex mixture of a high variety of molecules is the result in the oral cavity, frequently called “mixed saliva” and/or “whole saliva” in the scientific literature.

Whole saliva is a major determinant of the environment on all the oral surfaces. On tooth surfaces saliva plays an important role in acquired pellicle formation, which is a thin (*ca.* 0.5–1 μm) layer of several salivary proteins with calcium hydroxide binding properties [1,2]. Acquired pellicle plays a major role in crystal growth homeostasis of the teeth, and in physico-chemical defense of tooth surfaces. Acquired pellicle plays a major role in bacterial adhesion (and colonization) on tooth surfaces which may disadvantageously lead to caries formation and periodontal inflammation (especially in the absence of proper oral hygiene) [1,2]. Acquired pellicle, however, may also be considered as an important tool for advantageous surface exclusion of transient pathogen microbes entering the mouth (*i.e.*, infectious viruses such as influenzas). Besides defense of tooth surfaces, saliva plays an important role in physico-chemical as well as immune defense of the oral (and upper gastro intestinal) mucosal surfaces (via both direct antimicrobial action, as well as agglutination or surface exclusion of microbes). Saliva also plays important role in the healing of several mucosal lesions, wounds and ulcers as well [1,2].

### 1.2. The Innate and Acquired Oral Immunity

There are numerous defense proteins present in the saliva. Some of these defense proteins, like salivary immunoglobulins, and salivary chaperokine HSP70/HSPA, are involved in both innate and acquired immune activation [2–4]. Salivary cationic peptides and other salivary defense proteins, like lysozyme, BPI, BPI-like and PLUNC proteins, salivary amylase, cystatins, prolin-rich proteins, mucins, peroxidases, statherin (and others), are primarily responsible for innate immunity [1,2]; notwithstanding that, many of them also exert immune activator and/or immune modulator properties. Importantly, many of these molecules are present in rather low concentrations in whole saliva; however, it should be considered that their effects are cumulative and/or synergistic, resulting in an efficient molecular defense network of the oral cavity [1,2,5]. It should be also considered that local concentrations of these proteins near the mucosal surfaces (mucosal transudate), periodontal sulcus (gingival crevicular fluid) and oral wounds and ulcers (transudate) may be much greater, and in many cases reinforced by immune and/or inflammatory reactions of the oral mucosa [3,4]. Binding of salivary defense proteins onto the tooth and/or mucosal surfaces may also lead to an enrichment [6–8] of these proteins on tooth [7] and mucosal surfaces [8]. It is also likely that their basic properties are not altered via such binding [7–9]; moreover, surface binding may improve their resistance against proteolytic degradation via microbial proteases [10]. Besides the above, there may be an enrichment of defense proteins through participation in the formation of salivary micelles as well [11,12]. Concentration of salivary defense proteins may also be rather high near those cells, producing several defense proteins (*i.e.*, mucosal epithelial cells and neutrophil granulocytes entering the oral cavity).

### 1.3. Expected Network Action of the Salivary Defense System

As it was pointed out above, most (if not all) salivary defense proteins may enrich concentrations to “efficient” levels in certain locations in the oral cavity [1–4], notwithstanding that many of them are found in the whole saliva in less-than-efficient concentrations [13]. In these locations, a “single hit” type of action (one type of agent affects the target [14]) of salivary defense proteins may occur. However, salivary proteins (including cationic peptides) may also provide antimicrobial defense, even if they are present in concentrations that are lower than efficient. In this case, a network type, “multi-hit” approach may be expected. In this case, several kinds of salivary defense proteins affect the targeted microbe at the same time. Although in this case a certain salivary protein may induce a “partial knockout” [14] of the targeted microbe only, their network type “acting together” at the same time may lead to efficient elimination of the target.

We may expect five primary defense networks of salivary proteins in whole saliva. The first network may be responsible for microbial agglutination and/or surface exclusion. This network may include those salivary proteins and peptides which bind bacteria (microbes) and also bind to either oral surfaces [6–9] or each other [11,12] or both. The second network may be expected to be responsible for lysis of microbial membranes. This network primarily targets bacteria, and are likely to include salivary cationic peptides and lysozyme (the latter is likely to improve the efficiency of cationic peptides, as described later). The third and fourth networks may be responsible for antifungal [15,16] and antiviral [17] properties of the saliva respectively. These networks may include numerous salivary proteins exerting various antifungal [15,16] or antiviral [17] properties respectively. Finally, a fifth, immune regulatory network of salivary proteins may also be expected. This network is likely to include all those salivary proteins which exert immune activator/modulator properties. This network may be important for the fine-regulation of the local action of the mucosal immune system.

### 1.4. Multifunction Character of Salivary Defense Proteins

In the following paragraphs, the most important molecular participants (*i.e.*, defense proteins and peptides) responsible for the maintenance of the complex oral innate and acquired immune defense system will be introduced in detail. During their introduction it will be pointed out that most of these proteins (and peptides) are multifunctional [1,2,5], and that their action may even overlap in several cases [1,2]. This multifunctional character of salivary defense proteins also refers to their ability to act together in a “multi hit” network type approach (as expected above).

## 2. Salivary Antibodies

### 2.1. Major Classes of Salivary Antibodies

Salivary antibodies act in the first line of defense by performing immune exclusion of antigens in the saliva, in the mucus layer on the epithelial surfaces [18] and in the acquired pellicle on the tooth surfaces. They are constitutively excreted into the saliva and the oral cavity. There are two major antibody classes in human saliva, namely secretory IgA (sIgA) and IgG [1,2,18]. Secretory IgA is primarily of salivary gland and (in fewer amounts) of mucosal cell origin [18–20] and primarily dimeric (although in some cases may be polymeric) [18]. Salivary IgG is monomeric and either serum derived or produced by local plasma cells [18]. There is also a small fraction (15% of total salivary IgA) of monomeric (non-secretory) IgA in the saliva which is also either serum-derived or produced by local plasma cells, similarly to IgG [18]. Although the majority of salivary antibodies belong to IgA (90–98%) and IgG (1–10%) [1,2] classes, there are some very small fractions of IgM, IgD and IgE antibodies in the saliva as well [2,18].

Serum derived IgG and monomeric (non-secretory) IgA mainly enter the oral cavity via the gingival crevicular fluid [1,2,18], but also via mucosal transudate and via ultrafiltration through the salivary gland acini [1,2,18]. Similarly, local plasma cell produced monomeric (non-secretory) IgA and IgG may also enter the oral cavity via mucosal transudate and via acinar ultrafiltration [1,2,18]. Importantly, the numbers of antibodies transported through premised routes may strongly be influenced by certain oral conditions, such as gingival and mucosal inflammations, as well as by the integrity of the mucosal and acinar epithelial barrier [1,2,18].

### 2.2. Production of Salivary Secretory Antibodies

The initial stimulation of secretory immunoglobulin expressing B-cells (capable of differentiating into dimeric/polymeric antibody producing plasma cells) takes place in mucosa associated lymphoid tissues (MALT), such as gut-associated lymphoid tissues (GALT) and nasopharynx-associated lymphoid tissues (NALT) [18,21]. Notwithstanding that activated B-cells can migrate from GALT to salivary glands and are able to induce significant salivary secretory immunoglobulin response [18,21], in general, intestinal immune induction seems to be not so well reflected in the salivary secretory immunoglobulin system [18]. Thus, NALT tissues (*i.e.*, adenoids and palatine tonsils of the Waldeyer’s ring), orchestrating regional immune functions against both airborne and alimentary antigens, are likely to be the primary inductive sites for B-cells (capable of differentiating into dimeric/polymeric antibody producing plasma cells) destined to the salivary glands [18] and oral mucosa.

Salivary secretory immunoglobulins, namely secretory IgA (sIgA), and the very small amount of secretory IgM (sIgM), are produced by specific plasma cells residing primarily in the salivary glandular stroma and, in smaller numbers, also in the oral mucosa [19,20]. These plasma cells release mainly dimeric (but also some polymeric) IgA molecules and a few pentameric IgM molecules, all of which are stabilized by an incorporated 15 kDa J chain [18]. Both dimeric/polymeric IgA and pentameric IgM molecules are internalized from the interstitium and exported to the saliva by acinar cells (serous-type) and ductal cells (intercalated type) of minor and major salivary glands via a common salivary gland epithelial transport mechanism utilizing the polymeric immunoglobulin receptor (pIgR) and its major chain, the secretory component (SC) [18]. Importantly, secretory immunoglobulin A (and may be also sIgM) is constitutively excreted into the saliva even in the absence of stimulation [22]. A similar transport mechanism from the mucosal interstitium to the oral mucosal surface is also very likely in the case of oral mucosal epithelial cells [19,20]. However, the amount of oral mucosal epithelial transport seems to be much lower, compared to that of salivary glands [19].

### 2.3. The Secretory Component of pIgR

The secretory component (SC) of the polymeric immunoglobulin receptor (pIgR) exhibits strong affinity for the J chain of dimeric and polymeric IgA and pentameric IgM [18]. It appears to have similar affinity for both subtypes of polymeric IgA (IgA1 and IgA2; the latter is more resistant to certain bacterial proteases [23]); therefore both are equally well exported by the pIgR (containing SC) into the secretion [18]. The polymeric immunoglobulin receptor (pIgR) is a carbohydrate-rich 100 kDa glycoprotein constitutively expressed on the basolateral surface of glandular epithelial cells [18] and mucosal epithelial cells [20]. At the basolateral epithelial surface, the J chain of dimeric/polymeric IgA and pentameric IgM binds to SC (of pIgR), leading to a selective “lock and key” initialization of the molecular transport [18]. This molecular transport starts with the internalization of SC bound (pIgR bound) dimeric/polymeric antibodies at the basolateral cell surface and finished by the exocytosis of SC binding antibodies at the apical cell surface into the glandular lumen (or into the oral cavity in the case of mucosal epithelial transport). The process of exocytosis is coupled with the cleavage of the secretory component (SC) from the pIgR. Thereafter, the 80 kDa SC (former part of the 100 kDa pIgR) remains incorporated into the sIgA and sIgM antibodies permanently, endowing the secretory immunoglobulins with resistance against proteolytic degradation [18].

The expression of SC (pIgR) can be enhanced by cytokines [24] and excess, unoccupied pIgR (SC) is released from glandular cells (in the same manner by proteolytic cleavage as above), resulting in the presence of free secretory components (also of 80 kDa) in the saliva [18]. A similar release of free SC from those mucosal epithelial cells that discharge secretory immunoglobulins may also be expected. Importantly, free SCs exert several innate defense functions in the mouth, such as inhibition of epithelial adhesion of certain bacteria via (nonspecific scavenger type [25]) binding of bacterial fimbrial adhesins [26], as well as neutralization of certain bacterial toxins [27]. Immunoglobulin bounded SC exerts mucophilic properties and significantly contributes to the proper anchorage of sIgA to the mucus layer lining the mucosal surface [25,28]). As a non-specific microbial scavenger [25], immunoglobulin bounded SC may also play a role in the phenomenon that secretory antibodies show much better agglutinating properties than monomeric antibodies.

### 2.4. Antigen Binding, Agglutination and Surface Exclusion

The primary function of salivary (secretory and other) immunoglobulins is to inactivate parasites: bacteria, fungi and viruses (as well as certain microbial toxins) via binding and/or agglutination of such particles [18,29–31]. Such binding and/or agglutination may prevent mucosal adhesion of microbes and their toxins [30,31] and can lead to clearance toward the stomach, resulting in a consequent acidic digestion [30]. Surface immune exclusion (fixation to surfaces and immobilization until elimination) of the pathogens within the oral cavity via anchorage of secretory immunoglobulin-binding antigens to the superficial mucous layer lining the mucosal surface is another important mechanism to prevent invasion of the underlying tissues [25,32]. Since immunoglobulins are usually participants of the acquired pellicle of the teeth and promote microbial adhesion onto tooth surfaces, premised processes of immune exclusion are also very likely to occur on the surfaces of teeth. Surface immune exclusion seems to be an efficient and advantageous defense mechanism against those pathogenic microbes which are not dangerous to the oral tissues locally (*i.e.*, influenza viruses, bacteria responsible for enteritis or pneumonia *etc.*) [25,32]. However, it should also be considered that surface immune exclusion may also lead to the oral appearance and existence of immobilized microbes being strongly pathogenic locally (*i.e.*, cariogenic or periodontopathogenic bacteria *etc.*). Surface immune exclusion-related immobilization of microbes on tooth surfaces may also promote disadvantageous microbial biofilm formation on teeth (*i.e.*, dental plaque) in the absence of proper oral hygiene [1,2].

### 2.5. Phagocytosis, Antigen Presentation, Degranulation and Cytokine Production

Antigen binding and agglutination may also lead to phagocytosis (followed by antigen presentation and lysis) [18,30], as well as degranulation and cytokine production in the presence of immune-competent cells [33]. The oral mucosal surface is extensively populated by antigen presenting cells (*i.e.*, Langerhans and dendritic cells) and there is also a considerable flux of neutrophil granulocytes through the gingival sulcus into the saliva even in the healthy. Since both dendritic cells and neutrophil granulocytes have immune activating IgA receptors [33] on their surfaces, premised immune-cell coupled defense functions of salivary IgA (and maybe also other immunoglobulins) are likely to be active to maintain immune surveillance, also in healthy subjects. In the case of oral mucosal lesions and wounds, other immune-competent cells are certainly also available for a much more active immune defense reaction.

### 2.6. Antibody Catalyzed Ozone Formation

In the presence of reactive oxygen intermediate (ROI)-producing neutrophil granulocytes [34], salivary antibodies can catalyze ozone formation leading to efficient microbial killing [35–37]. During this process, antibodies kill microbes by catalytically converting a less toxic ROI (namely singlet dioxygen supplied by the neutrophils) to a mixture of hydrogen peroxide and ozone [38]. This strongly improves the efficiency of killing, because, on the one hand, ozone is a very powerful oxidant, and on the other, no microbial enzymes are known to catabolize ozone [38]. Importantly, all immunoglobulins, regardless of their source or antigenic specificity [35,36], are able to induce and catalyze such ozone formation; thus, antibodies bridge innate and adaptive immunity in this respect [38]. Interestingly, it is not only intact antibodies that are able to catalyze the reaction, but also their fragments [39], as well as certain single amino acids (namely tryptophan, methionine, cysteine and histidine) [40].

### 2.7. Other Functions of Dimeric/Polymeric Antibodies

Besides the above, there are some other dimeric/polymeric immunoglobulin-related mechanisms which also contribute to mucosal immune defense against antigens (primarily viruses and microbial toxins) [25,32]. For example, polymeric immunoglobulin receptor (pIgR) is able to bind and internalize antigen loaded dimeric/polymeric immunoglobulins on the basolateral epithelial cell surfaces and exocytose the antigen-immunoglobulin complex in the apical side of the cell into the glandular lumen (or into the oral cavity in case of mucosal epithelial transport) [25,32]. It may also occur that the pathogen is neutralized in the epithelial cell during this antibody coupled pIgR driven transport [25,32]. Similarly, dimeric/polymeric immunoglobulins being transported through the epithelial cells (during pIgR mediated transcytosis as above) can bind intracellular antigens (*i.e.*, invading viruses or bacterial lipopolysaccharide/LPS/toxins) and thereby perform intracellular antigen neutralization and clearance which inhibits epithelial cell damage and prevents the appearance of inflammation [32,41]. Premised pathways suggest important clearing mechanisms targeting (and eliminating) those antigens already invading the epithelial cells and/or the underlying glandular and mucosal tissues (*i.e.*, glandular stroma and lamina propria mucosae) [25,32].

Although salivary IgA (including both secretory and monomeric forms) does not activate a complement system by direct means, salivary IgG type antibodies, however, are complement activating [32]. Salivary IgG-induced complement activation may occur in healthy subjects near the gingival sulcus, because the composition of the gingival crevicular fluid is very similar to serum transudate (even in healthy subjects) containing also the complement system. In the case of oral mucosal lesions and wounds, salivary IgG-induced complement activation may also occur in the injured regions of the oral mucosa.

## 3. Salivary Chaperokine HSP70/HSPAs

### 3.1. The HSP70/HSPA Protein Family

The HSP70/HSPA type proteins are 70 kDa major molecular chaperones and cytokine chaperokines [42] of most cells and tissues, extracellular and interstitial fluids, blood, synovial fluids, and also secreted body fluids, like saliva [43–45]. Extracellular HSP70/HSPAs reveal cytoprotective properties through cell surface association, which may be followed by internalization. Extracellular HSP70/HSPAs are also involved in a number of physiological and pathological events, including modulation of cytokine release, immunity, and the modulation of neuronal function [42,45]. In addition, HSP70/HSPAs are able to enter the bloodstream, and possess the ability to act at distant sites of the body as an ancestral danger signal triggered by cell injury, immune-inflammatory reactions, and physical or behavioral [44] stress of the organism.

As mentioned above, the presence of HSP70/HSPAs in human saliva was also demonstrated [43,44]. It is very likely that both constitutively expressed and stress inducible forms of HSP70/HSPA proteins are also present in human saliva (see also below) [43,44,46]. The presence of HSP70/HSPA proteins in the oral environment refers to the extracellular functions of Hsp70/HSPA proteins that should also be considered in relation with oral defense mechanisms.

### 3.2. Origin of Salivary HSP70/HSPAs

Salivary glands are among the main sources of HSP70/HSPAs in the saliva [3,43,44]. Although it has not yet been investigated in detail, it is very likely that HSP70/HSPAs of glandular origin are a mixture of both constitutively expressed and stress inducible forms of HSP70/HSPA [3,43,44]. The level of salivary HSP70/HSPAs shows large differences between subjects in both mixed (whole) saliva and cannulated parotid duct saliva [46]. Within a subject, there are also differences, maybe because of the prompt inducibility of salivary HSP70/HSPAs via several stimuli [43,44]. It is very likely that HSP70/HSPAs are not secreted due to the classical secretory exocytotic process of the acinar cells [43,44]. The transport of HSP70/HSPAs may involve passive transport via the salivary glands (and their ducts) from blood serum [3,43,44], and/or the possibility of small capacity active transport from the striated duct cells of human salivary glands [3,43,44]. An alternative transport of HSP70/HSPAs may also occur either through lipid rafts or through exosomes [3,43,44].

Besides salivary glands, there are also other important sources of salivary HSP70/HSPAs, such as mucosal cells [3,43,44], gingival crevicular fluid [3,43,44], the oral mucosal transudate [2,4], and intraoral bleeding (*i.e.*, bleeding periodontal pockets, wounds, ulcers) [2,4]. There are also identified bacterial, fungal or parasitic homologues of HSP70/HSPA proteins in the oral cavity. Their sources are oral bacteria and other oral microbes [2,4,46].

### 3.3. Binding of Bacteria, Agglutination and Surface Exclusion

Salivary HSP70/HSPAs may entrap and agglutinate bacteria [46]. Recent data has indicated that salivary HSP70/HSPAs bind both gram-positive (*Streptococcus mutans* and *mitis*) and gram-negative (*Escherichia coli*) bacteria [46–48]. Since HSP70/HSPAs are known to be able to form dimers and oligomers, the agglutinating function of salivary HSP70/HSPAs could be efficient. It is also possible that HSP70/HSPAs occur in micelles and/or in smaller homo/heterotypic complexes, which are also known to enhance agglutination in saliva [11]. Importantly, salivary HSP70/HSPAs also bind hydroxyapatite—the major inorganic component of tooth surfaces [46]. Therefore, it is likely that salivary HSP70/HSPAs may play a role in the acquired pellicle formation followed by bacterial adhesion on tooth surfaces. The capability to take part in the acquired pellicle formation and to bind bacteria refers to the facility of salivary HSP70/HSPAs bacteria to colonize tooth surfaces, which may lead to dental caries and periodontal inflammation. On the other hand, binding of both tooth surfaces and bacteria may also lead to surface exclusion of those bacteria, which are not harmful locally but may be pathogenic for the whole organism.

### 3.4. Immunological Defense Mechanisms

Three major facets of immune activation have been described for salivary HSP70/HSPAs [3,43,44]. The first facet of immune activation involves released extracellular HSP70/HSPA as an ancestral danger signal of cellular stress, death or lysis. Importantly, both uncomplexed (“free”) and membrane bound (lipid rafts, exosomes) HSP70/HSPAs were shown to express such danger signal properties [49]. The immune activation here is very similar to that of bacterial lipopolysaccharides (LPS) and the effects of LPS and extracellular HSP70/HSPAs seem to be additive [49]. HSP70/HSPAs as danger signals may induce the release of proinflammatory cytokines from several immune cells (*i.e.*, monocytes, dendritic cells, macrophages, T lymphocytes), release of NO from macrophages, activation of NK cells and activation of complement via an antibody-independent alternative pathway [49–51]. HSP70/HSPA proteins also act as cytokines and chemikones in the presence of immune cells. These facets of immune function could be effective in the case of oral mucosal lesions (*i.e.*, oral wounds and ulcers), because inflammatory serum exudate containing a high amount of complement system, immunoglobulins, immune/inflammatory mediators as well as PMN leukocytes, and monocytes/macrophages are usually also present on the surface of oral lesions [3,4,46].

The second facet of immune system activation involves complexes of extracellular HSP70/HSPA proteins and other peptides. Because of the chaperoning ability, uncomplexed HSP70/HSPA binds other peptides, and as complex induced receptor-mediated uptake into antigen-presenting cells (*i.e.*, macrophages, Langerhans and dendritic cells) to cross-present this complex as an antigen (coupled with MHC-I or MHC-II molecules) to cytotoxic T cells and NK cells [52]. This mechanism is important in the defense against bacteria (and other microbes), and also as an initiator of immune defense against tumor cells and virus-infected cells. Since oral mucosa (especially non-keratinized parts) is extensively populated by antigen-presenting Langerhans and dendritic cells [53] (from which Langerhans cells are properly oriented to “sample” the oral fluids with their dendrites toward the surface [53,54]), these facets of immune function can be efficient in the mouth.

The third facet is based on the recent finding that HSP70/HSPA proteins exert an opsonizing effect on bacteria, which activate the killing activity of polymorphonuclear neutrophil granulocytes [47,48]. Although there is a considerable flux of neutrophils through the gingival sulcus into the saliva, even in the healthy, this function may be especially effective under inflammatory conditions (*i.e.*, gingival inflammations) and in the case of oral lesions (*i.e.*, ulcers, wound healing) [46].

### 3.5. Further Defense Functions of Salivary HSP70/HSPAs

Besides the above, there may be other defense functions of salivary HSP70/HSPAs, based on the known cytoprotective effects of extracellular HSP70/HSPA proteins [55,56]. The cytoprotective effects seem to be based on three different mechanisms. Aspecific binding of Hsp70/HSPA on mucosal cell surfaces [55] may lead to surface defense against toxins [55]. A more specific adhesin-type binding to sulfoglycolipid structures of mucosal cells [57] may prevent bacterial colonization of mucosal surfaces through occupying mucosal binding sites of HSP70/HSPA related bacterial adhesins. Surface receptor binding of HSP70/HSPAs may also occur, which is mostly followed by internalization [45,56]. To date, a decrease in cell apoptotic and necrotic liability [58] and the release of several cytokines [59,60] are the most important proven mechanisms of HSP70/HSPA receptor-induced cytoprotection.

Further, another defense mechanism has also been hypothesized [46], based on the finding that fungicidal activity of salivary Histatin-5 (see also below) is initiated by binding to surface HSP70/HSPA homologues of *Candida albicans* (Ssa1p, Ssa2p), followed by internalization and later cell death [61,62]. This finding may indicate that Histatin-5 may bind human salivary HSP70/HSPA too. Although there is no evidence that such a salivary HSP70/HSPA-Histatin-5 complex would also be able to enter and destroy *C albicans*, this possibility should still be considered rather than simply excluded [46].

## 4. Cationic Peptides

### 4.1. Defensins

Defensins are “prototype” of cationic peptides. They are characterized by a “hairpin-like” globular structure stabilized by three intramolecular disulfide bridges linking six cysteine amino acids [54]. Based on the pattern of cysteine-pairing, two main subfamilies are distinguished, namely the α-defensins and the β-defensins [54,63]. Salivary α-defensins (HNP1, HNP2, HNP3, HNP4) are produced by neutrophil granulocytes [13,63], whereas salivary β-defensins (hBD1, hBD2, hBD3, hBD4) are produced by mucosal cells [13,63,64]. Besides whole saliva, both α- and β-defensins are also present in the gingival crevicular fluid [13]. Both α- and β-defensins show broad antibacterial activity, based on their cationic peptide character [13,63]. Their first interaction with bacteria is typically mediated by their positive net charge. In contrast to eukaryotic cells carrying no or little net charge on the outer leaflet of their cell membrane, outer leaflets of bacterial cell membranes are typically negatively charged [63]. Cationic peptide defensins adsorb via electrostatic forces onto the negatively-charged bacterial cell membrane leading to their subsequent aggregation and integration into the lipid bilayer [63,65]. Integration of defensins into the bacterial membranes results in the formation of ion channels, transmembrane pores, membrane leakages and membrane rupture [65], leading to the destruction of the bacteria. How cationic peptides (including defensins) manage to get across polysaccharide bacterial capsules (if any) and peptidoglycan layers of bacterial membranes is far not fully understood yet, however, digestion of peptidoglycans via salivary lysozyme is likely to be an important assistance to get across the peptidoglycan layers. Importantly, antibacterial activity of most defensins (but not of hBD3) can be neutralized by higher salt concentrations (*i.e.*, 100 mM monovalent or 2 mM divalent cations) [66], which may also be present transiently in stimulated (*i.e.*, chew and/or taste) saliva [2]. Besides their broad antibacterial activity, defensins also exert antifungal and antiviral properties [17,63]. An antifungal effect of β-defensins is likely to occur via binding of fungal HSP70-type surface proteins (in particular: Ssa1p of *Candida albicans* [15]) may be followed by an internalization process similar to that of histatin-5-dependent antifungal activities (see below). Defensins also exert various immune activator and modulatory activities, including induction of certain cytokines and chemoattractivity for immature dendritic and memory T-cells [63].

### 4.2. Histatins

Histatins are small histidine-rich cationic peptides ranging in size from 7 to 38 amino acids. Histatins are secreted by the parotid gland as well as the sublingual and submandibular glands [13,67]. There are about a dozen histatins (HRPs), of which the most important are: histatin-1, histatin-2, histatin-3 and histatin-5 (this last one is a proteolytic cleavage derivative of histatin-3 [13]) accounting for 85–90% of this family. Histatins exert broad-spectrum antibacterial as well as antifungal properties [63,68]. Histatins also show antiviral properties [17]. As cationic peptides, histatins adsorb via electrostatic forces onto the negatively charged bacterial cell membranes leading to subsequent histatin aggregation followed by their integration into the lipid bilayer [63,65]. Their integration into the bacterial membrane is likely to lead to the formation of ion channels, transmembrane pores, membrane leakages and membrane rupture [65], causing destruction of bacteria. Histatins also bind and complex Cu^2+^ and Ni^2+^ ions [63,69], leading to the elimination of metal ions and the consequent inhibition of enzymes, their cofactors and microbial growth [63,69,70]. Histatins (especially histatin-5) also exert efficient activities against fungi (in particular: against the yeast *Candida albicans*) [68]. This antifungal activity is initiated by binding histatin-5 to certain HSP70-type surface proteins (Ssa1p, Ssa2p) of *Candida albicans*, followed by internalization of histatin-5 and consequent cell death [61,62]. HRP5 was also shown to inhibit a trypsin-like protease of *Bacteroides gingivalis* [71]. Histatins (especially histatin-1) are also incorporated into the acquired pellicle on tooth surfaces [9], and therefore may play a role in bacterial colonization (and/or surface exclusion of bacteria) on tooth surfaces. On the other hand, histatin-1 was also shown to competitively inhibit the absorption of high molecular weight glycoproteins (HMWGPs) to tooth surfaces, and therefore may inhibit adhesion of HMWGP-binding cariogenic bacteria (*i.e.*, *S. mutans* [72]) onto tooth surfaces [72]. Histatins (especially histatin-2, but also histatin-1 and histatin-3) were also identified as highly important wound closure stimulating factors of human saliva [68]. HRPs also inhibit (precipitate) tannins, a widespread occurring phenolic plant compound (flavonoid) with unpalatable astringent and protein precipitating properties [73].

### 4.3. Lactoferrin

Lactoferrin is an iron-binding cationic glycoprotein of 80 kDa which is present in most exocrine secretions, including saliva [63]. Major sources of lactoferrin in saliva are the salivary glands, neutrophil granulocytes entering the oral cavity [63], and the mucosal epithelial cells [13]. Lactoferrin is also present in the gingival crevicular fluid, which is also a significant source of lactoferrin present in the saliva [13]. Lactoferrin is active against bacteria, fungi, parasites and viruses [13,63]. Lactoferrin has a positive net charge and this cationic property seems to be an important factor which may lead to the binding and destruction of microbial cell membranes [63] as detailed above. Proteolytic cleavage generates smaller cationic peptide derivatives of 25 amino acids (or fewer) which exerts strong (iron-binding independent) bacteriolytic properties [74,75], very likely via electrostatic force-derived membrane adhesion and accumulation, followed by membrane destruction, as is usual in the case of cationic peptides (see above). Smaller derivatives seem to display pronounced antifungal activity as well [74]. Besides its cationic peptide activity, lactoferrin is a known scavenger of Fe^3+^ ions [13]. It binds and sequestrates iron, depriving microorganisms (*i.e.*, bacteria, fungi and parasites) of the iron that is essential for their growth [63]. Lactoferrin also binds bacterial fimbrial adhesins, and therefore inhibit epithelial adhesion of certain bacteria [26]. Antiviral activity of lactoferrin [76] is expected to be based on binding (and blocking) of certain host cell glycosaminoglycans used by viruses for adsorption [63]. Lactoferrin may also neutralize viruses by direct binding [63]. Immune modulatory and anti-cancer activity of lactoferrin also seems likely [63].

### 4.4. Cathelicidins (LL-37)

Cathelicidins are characterized by a conserved *N*-terminal domain that is proteolytically cleaved to generate the active peptide [13,63,66]. The human cathelicidin is an 18 kDa cationic protein referred to as hCAP-18 [63,77]. Its most important active derivative is a 16 kDa peptide referred to as LL-37 [13,63]. LL-37 is an α-helix type cationic antimicrobial peptide, which may be further cleaved, resulting in smaller derivatives (RK-31, KS-30) of even higher antimicrobial activity [63,77]. Salivary LL-37 is likely to originate primarily from neutrophil leukocytes [13]. LL-37 is also present in the gingival crevicular fluid [13]. Since LL-37 (and its derivatives) are cationic peptides, their antibacterial effect is based on their aggregation onto microbial membranes and destruction via formation of ion channels, transmembrane pores, membrane leakages or membrane rupture [65]. LL-37 also binds and neutralizes bacterial lipopolysaccharides (LPS) [13]. Further, LL-37 and its derivatives are likely to play a role in the re-epithelialization of wounds and ulcers [78] in the oral cavity. LL-37 and its derivatives are also expected to exert immune activator and immune modulator properties [63].

### 4.5. Secretory Leukocyte Proteinase Inhibitor

Secretory leukocyte protease inhibitor (SLPI) is an 117 kDa (107 amino acids) serine protease inhibitor, that controls excessive proteolysis caused by proteases (*i.e.*, elastase, cathepsin G) of neutrophil granulocytes [13,63,79]. Therefore, SLPI is also referred to as antileukoprotease (ALP). Secretory leukocyte protease inhibitor (SLPI) present in saliva is produced by keratinocytes of the oral mucosa [13,63,80] as well as by neutrophil granulocytes entering the oral cavity [63]. SLPI is a non-glycosylated, basic, single-chain, cysteine rich cationic polypeptide [63]. Because of its cationic character, it is likely to act as a cationic peptide, aggregating onto microbial membranes and destruction of via the formation of ion channels, transmembrane pores, membrane leakages or membrane rupture. SLPI exerts antimicrobial activity against both bacteria (*P. aeruginosa*, *S. aureus*) and fungi (*C. albicans*) [13,81], and also exerts antiviral properties [80].

### 4.6. Adrenomedullin

Adrenomedullin is a pluripotent hormone-like cationic peptide of 52 amino acids. It is present in the gingival crevicular fluid, glandular saliva and whole saliva [13,82]. It is also very likely that oral epithelial cells excrete adrenomedullin into the saliva [13,82,83]. Because of its cationic peptide character, adrenomedullin is able to kill bacteria [84,85] via aggregation onto bacterial membranes and destruction via the formation of ion channels, transmembrane pores, membrane leakages or rupture. It is also able to prevent bacterial growth (*S. aureus*) via generation of abnormal septum formation during cell division [85]. Adrenomedullin also dose dependently inhibits growth of several other bacteria via an unknown mechanism [82]. Proteolytic cleavage of adrenomedullin may lead to derivatives of even higher antimicrobial activity [85].

## 5. Further Innate Defense Proteins of Saliva

### 5.1. Lysozyme

Lysozyme is a small (145 kDa) protein present in body fluids, including saliva. Salivary lysozyme is produced by the salivary glands (highest level was found in the sublingual saliva [86]) and also by neutrophil granulocytes entering the mouth [63]. It is also present in the gingival crevicular fluid [13]. Lysozyme exerts muramidaze activity via hydrolysis of the β-1,4-glycosidic bonds between *N*-acetylmuramic acid and *N*-acetyl-d-glucosamine of bacterial cell wall peptidoglycan. Lysozyme mainly kills gram-positive bacteria that damages surface-exposed peptidoglycan. The reduced susceptibility of most gram-negative species may be because of the outer membrane of these bacteria that shields the peptidoglycan layer from the environment [63]. Digestion of peptidoglycan structures via salivary lysozyme may also assist antimicrobial cationic peptides of saliva to get across peptidoglycan layers of bacterial membranes. It may also be hypothesized that cationic peptides may produce membrane leakage of the outer membrane of gram negative bacteria to assist lysozyme to reach the peptidoglycan layer of these bacterial membranes. Importantly, the killing of bacteria by lysozyme is, in many cases, largely independent of its enzymatic activity [87,88]. In these cases, the membrane permeabilizing property of lysozyme is likely to play a role [88]. This non-enzymatic antimicrobial property of lysozyme seems to be active against both gram-positive and gram-negative bacteria [88] as well as fungi [88,89]. Besides the above, lysozyme also exerts antiviral properties [90] and may also induce lysis of tumor cells [91]. Lysozyme also binds bacterial lipopolysaccharide (LPS) [92], a bacterial surface structure and bacterial toxin, frequently responsible for tissue destructive inflammatory reactions. It is also expected that lysozyme can influence human granulocyte and lymphocyte function, and may inactivate viruses [93].

### 5.2. BPI, BPI-like and PLUNC Proteins

These proteins belong to the same lipid-binding protein family and show more or less similar molecular structures. Bactericidal/permeability increasing protein (BPI) is a 55 kDa cationic protein. Primary sources of salivary BPI are neutrophil granulocytes [63] and the epithelial cells of the oral mucosa [63]. BPI exerts bactericidal, endotoxin neutralizing and opsonic properties. BPI shows high affinity to the lipid-A moiety of bacterial lipopolysaccharide (LPS) structures. The antibacterial and endotoxin-neutralizing activity of BPI belongs to its LPS-binding *N*-terminal domain, whereas the opsonic property belongs to its *C*-terminal domain [63]. The most important representative of BPI-like proteins (bactericidal/permeability, increasing protein-like proteins) in the saliva is the parotid secretory protein (PSP) [13]. PSP is secreted by the salivary glands and also by the keratinocytes of the oral mucosa [13,94,95]. This protein is likely to be bacteriostatic, bind bacterial LPS and promote agglutination of bacteria [13]. Salivary PLUNC proteins (proteins of palate lung and nasal epithelial clone family) are primarily produced by the major and minor salivary glands [63,96]. There are eight functional PLUNC proteins in humans, which can be divided into two subgroups, such as short type S-PLUNC proteins (SPLUNC-1, SPLUNC-2, SPLUNC-3) and long type L-PLUNC proteins (LPLUNC-1, LPLUNC-2, LPLUNC-3, LPLUNC-4, LPLUNC-6) [63]. Short type PLUNC proteins consist of only one domain corresponding to the LPS-binding *N*-terminal domain of BPI, whereas long type PLUNC proteins consist of two domains similar to the whole BPI molecule [63]. PLUNC proteins appear to be able to bind bacterial LPS similar to BPI [63]; however, PLUNC proteins are not likely to exert direct killing activity (they are likely to be bacteriostatic, similarly to PSP) [63]. PLUNC proteins are also likely to promote agglutination of bacteria and to modulate cytokine production [63].

### 5.3. α-Amylase

Salivary amylase is a highly abundant protein in saliva. The highest concentration of amylase was found in the saliva of the parotid gland and palatine minor salivary glands [86,97]. Eight isoforms contain asparagine-linked sugar chains and are around 61–63 kDa [6,98,99], whereas other eight isoforms do not have such chains are around 56–59 kDa [6,98,99]. The most widely-known function of amylase is endoglicosidase activity. Splitting the α-1,4-glicosidic bindings of several glycans, such as starch (amylopectin), amylase produces oligosaccharides (dextrin) disaccharides (maltose, isomaltose) and monosaccharide glucose. Beside its enzymatic activity, amylase also takes part in acquired pellicle formation on tooth surfaces [98,100,101]. Amylase also binds bacteria [92,98,101], including certain bacterial pili [102] which are important factors of bacterial adhesion. Thus, amylase promotes bacterial adhesion to the hydroxyapatite surfaces of teeth [100,101], which can lead to both advantageous surface immune exclusion on the one hand and disadvantageous adhesion of cariogenic or periodontopathogenic bacteria onto tooth surfaces on the other hand. In contrast, the binding of bacteria may also lead to the prevention of bacterial surface adhesion via saturation of bacterial adherence factors of “floating” planktonic bacteria (*i.e.*, like bacterial pili [102]) and consequent bacterial clearance towards the stomach [98] resulted in acidic digestion. It was also demonstrated that amylase performs a direct inhibitory effect on the growth of certain bacteria [30,98,103]. Amylase also binds bacterial lipopolysaccharide (LPS) [92], a bacterial surface structure and bacterial toxin, responsible, in many cases, for tissue destructive inflammatory reactions. Amylase may also exert virus inhibitory properties [104].

### 5.4. Cystatins

The human cystatin gene family contains 14 genes (including two pseudogens) from which seven cystatins are present in saliva [13], namely cystatin-A, cystatin-B, cystatin-C, cystatin-D, cystatin-S, cystatin-SA and cystatin-SN [13]. The highest concentration of cystatins was found in the submandibular saliva [86], but (at a much lower concentration) they are also present in the parotid saliva [105]. Cystatins are also present in the gingival crevicular fluid [106]. Cystatins are cysteine protease inhibitors that block the action of endogenous [13,105], bacterial [13] and parasitic protozoan proteases [105]. Cystatin-C and cystatin-S were shown to inhibit bacterial growth (*P. gingivalis*) [107]. Cystatin-SN and cystatin-S are present in the human-acquired enamel pellicle [9] and also bind bacteria [92] as well as bacterial lipopolysaccharides (LPS) [92], which are bacterial surface structures and toxins frequently responsible for tissue destructive inflammatory reactions. Cystatins also exert direct immunomodulatory properties [105]. They are also likely to exert certain antiviral effect [105].

### 5.5. Proline-Rich Proteins (PRPs)

Proline-rich proteins form a major fraction of salivary proteins (*ca.* 20–30 % of total), the molecular weight of acidic and basic PRPs is usually between 10–40 kDa, whereas large glycosylated PRPs have a molecular weight of 60–70 kDa [108]. Proline-rich proteins (PRPs) are highly phosphorylated proteins [73]. The major source of salivary proline-rich proteins (PRPs) are the salivary glands [2]; the highest concentration of PRPs was found in the parotid saliva [86]. PRPs are encoded by seven genes, and many of them are subsequently cleaved by proprotein convertases before secretion, consequently a large number (more than 20) PRPs exist. Acidic PRPs contain a longer and highly acidic *N*-terminal region, and a somewhat different repeat sequence, compared with basic PRPs [73]. Acidic PRPs exert calcium hydroxide-binding properties, and therefore participate in the formation of acquired pellicle (a thin, ≈0.5–1 μm protein layer) on the surface of teeth [2,9]. Basic PRPs are also present in the human-acquired enamel pellicle [9]. Acidic PRPs bind bacteria, basic PRPs bind fungi (e.g., *Candida albicans*) and viruses, whereas glycosylated PRPs bind bacteria and viruses that indicate the role of PRPs in the clearance towards the stomach and/or surface exclusion of these microorganisms [2,30,109]. PRPs are also potent precipitators of tannins, similarly to histatins [73].

### 5.6. Salivary Mucins

There are two major subtypes of mucins, namely the membrane associated and the secreted type [110]. In the oral cavity, membrane associated type mucins (*i.e.*, MUC-1) are primarily secreted by the oral mucosal epithelial cells [110]. These proteins mainly remain on the cell surface (in the mucus layer of mucosa) after secretion [110], and are primarily involved in the protection of epithelial surfaces [110]. There is also a much higher amount of secreted type mucins present in the oral cavity, from which salivary mucins MUC5b and MUC7 (older terms: MG1 and MG2) are the most important subtypes [2,86,111,112]. Salivary mucins are rather large and highly glycosylated proteins [112]. MUC5b has a molecular weight of higher than 1000 kDa and is composed of disulphide linked subunits [112]; whereas MUC7 is a monomer of approximately 180–200 kDa [112]. Salivary mucins are produced primarily by the submandibular gland and by the labial and palatinal minor salivary glands [86,111]. The highest concentration of salivary mucins was found in the saliva of sublingual glands (Mg1, MG2) and palatine minor salivary glands (high molecular weight mucins) [86]. Besides taking part in acquired pellicle formation on tooth surfaces (especially MUC5b type mucins [2,111]), salivary mucins cover all oral surfaces with an at least ≈10–22 μm (up to 40–60 μm [113]) thick layer [2,113]. In addition, MUC5b-type mucins form a hydrophilic viscoelastic gel (in low concentration) that causes a high viscosity matrix of saliva [2,113]. Salivary mucins, especially MUC7, have a high affinity to microorganisms, and entrap and agglutinate bacteria, fungi and viral particles [2,13]. MUC5b was also shown to exert antiviral properties [17]. Bactericidal and antifungal properties of a cationic peptide (MUC7 12-mer) derived from the *N*-terminal region of MUC7 was also reported [114].

### 5.7. Peroxidases

There are two principal components of the peroxidase system of saliva, namely lactoperoxidase and myeloperoxidase [115]. Lactoperoxidase is produced by the salivary glands, whereas myeloperoxidase is produced by neutrophil granulocytes entering the oral cavity [13]. Myeloperoxidase is also present in the gingival crevicular fluid [13]. Both lactoperoxidase (salivary peroxidase) and myeloperoxidase catalyze the oxidation of thiocyanate ions (SCN-) by hydrogen peroxide, leading to the production of a much more bactericidal [116] and fungicidal [116] agent, namely hypothiocyanite (OSCN-) [117]. Importantly, this function of salivary peroxidase seems to be facilitated by Duox-2, a homologue of the catalytic core (gp91) of NADPH oxidases [118,119]. Duox-1 is localized in the luminal plasma membrane of epithelial cells of major (terminal) collecting ducts of salivary glands [118, 119], and provides hydrogen peroxide (the most labile component of the system) for salivary peroxidase just prior to delivery into the oral cavity [118,119].

### 5.8. Statherin

Statherin is a 5.4 kDa (43 residues) tyrosine- glutamine- and proline -rich phosphoprotein. It inhibits precipitation of calcium phosphate salts from saliva, which is supersaturated with respect to these salts [120,121]. Furthermore, statherin not only inhibits the crystal growth of calcium phosphate salts, but also inhibits spontaneous (unseeded) precipitation from solutions supersaturated with respect to calcium phosphate salts (such as saliva). Further, statherin binds hydroxyapatite [9,30,120,121], indicating a possible role in acquired pellicle and dental plaque formation. On the other hand, statherin competitively inhibits the absorption of high molecular weight glycoproteins (HMWGPs) to tooth surfaces, and therefore may inhibit adhesion of HMWGP-binding cariogenic bacteria, including *Streptococcus mutans* [70]. Interestingly, statherin is likely to be enriched in the air interface of the saliva film present in the mouth [122]; which also may be an indication that the binding of bacteria by statherin more likely leads to aggregation and clearance towards the stomach than to surface adhesion. Besides its antibacterial properties, statherin also induces transition of hyphae (the most invasive form of the fungus) to yeast in *Candida albicans* [16]; indicating that statherin is likely to contribute to oral defense against fungi [16].

### 5.9. Salivary Agglutinin (SAG, gp-340)

Salivary agglutinin (SAG) is a scavenger-receptor cysteine-rich glycoprotein [123]. It is also referred to as lung glycoprotein-340 (gp-340) and also as protein deleted in malignant brain tumors (DMBT-1) [123]. In the saliva, SAG acts as a pattern recognition scavenger receptor, and as such, it binds a broad range of oral pathogens, including bacteria and viruses [123,124,125]. Similarly, SAG also binds salivary proteins, including IgA and mucin MUC5b [123]. Based on premised properties, SAG efficiently aggregates bacteria and viruses, and significantly increases their clearance from the mouth towards the stomach, leading to their acidic digestion [126]. Salivary agglutinin (SAG) is also present in the acquired pellicle on the tooth surfaces and in the mucous layer on the mucosal cell surfaces [126]. Thus, it may promote bacterial/viral adhesion onto oral surfaces [126], which may lead either to pathogenic invasion and/or the surface exclusion of pathogens. However, the majority of SAG is found soluble in saliva; therefore, surface related effects are of less importance in the oral cavity [126]. Besides its efficient antibacterial and antiviral properties [17,124,125], salivary agglutinin also shows certain immune activator/modulator activities [123].

### 5.10. Other Defense Proteins of Innate Immunity

There are likely to be numerous other defense proteins present in saliva from which at least calprotectin and three bacteria binding proteins should be mentioned. Calprotectin is a dimer of calgranulin-A and calgranulin-B with metal ion-binding properties [13]. Calprotectin inhibits microbial growth by acting as a divalent cation (*i.e.*, Mn^2+^, Zn^2+^) scavenger [127]. The major sources of salivary calprotectin are oral epithelial cells and neutrophil granulocytes [13] entering the oral cavity. Calprotectin is also present in the gingival crevicular fluid [128]. There are three bacteria and LPS-binding proteins, namely prolactin inducible protein (PIP), lipocalin (LCN) and submandibular gland androgen-regulated protein (SMR) which should also be mentioned. These proteins bind bacteria and bacterial lipopolysaccharide (LPS), which is a bacterial surface structure and bacterial toxin, frequently responsible for tissue destructive inflammatory reactions [92].

## 6. Conclusions

Whole saliva is a major determinant of the environment on all the oral surfaces. On tooth surfaces, saliva plays an important role in acquired pellicle formation, which in turn plays a major role in crystal growth homeostasis and physico-chemical defense of the teeth [1,2] as well as in bacterial adhesion (and colonization) to tooth surfaces which may lead to caries formation and periodontal inflammation [1,2]. Acquired pellicle, however, may also be considered as an important tool for advantageous surface exclusion of transient pathogen microbes entering the mouth. Saliva also plays an important role in physico-chemical as well as immune defense of oral mucosal surfaces (via both direct antimicrobial action and agglutination or surface exclusion of microbes). Saliva also plays an important role in the fine regulation (activation/modulation) of oral mucosal immune reactions, as well as in healing of several mucosal lesions, wounds and ulcers [1,2]. There are numerous defense proteins present in the saliva. Some of these defense proteins, such as salivary immunoglobulins and salivary chaperokine HSP70/HSPAs are involved in both innate and acquired immune activation [2,3,4], whereas cationic peptides and other salivary defense proteins are primarily responsible for innate immunity [1,2]. Notwithstanding that, many of these molecules are present in a rather low concentration in whole saliva [13]; local concentrations of these proteins near the mucosal surfaces, periodontal sulcus and oral wounds and ulcers (transudate) may be much greater. Moreover, in many cases their effect is reinforced by immune and/or inflammatory reactions of the oral mucosa [3,4]. Their local concentration may also be high on mucosal and tooth surfaces [6,7,8,9], in the salivary micelles [11,12] and near the immune cells entering the oral cavity. Their effects are also additive and/or synergistic, resulting in an efficient molecular defense network of the oral cavity [1,2,5]. In this latter case, a network type “multi-hit” approach may be expected, during which different kinds of salivary defense proteins affect a targeted microbe at the same time. Most salivary defense proteins (and peptides) are multifunctional [1,2,5] and their actions overlap in several cases [1,2], which serves as a good basis for this “multi hit” network type defense action.

## References

[1] Fábián T.K., Fejérdy P., Csermely P. (2008). Salivary genomics, transcriptomics and proteomics: The emerging concept of the oral ecosystem and their use in the early diagnosis of cancer and other diseases. Curr. Genomics.

[2] Fábián T.K., Fejérdy P., Csermely P, Begley T.P. (2008). Saliva in Health and Disease (Chemical Biology of). Wiley Encyclopedia of Chemical Biology.

[3] Fábián T.K., Fejérdy P., Nguyen M.T., Sőti C., Csermely P. (2007). Potential immunological functions of salivary Hsp70 in mucosal and periodontal defense mechanisms. Arch. Immunol. Ther. Exp.

[4] Fábián T.K., Gótai L., Beck A., Fábián T.K., Fejérdy P. (2009). The role of molecular chaperones (HSPAs/HSP70s) in oral health and oral inflammatory diseases: A review. Eur. J. Inflamm.

[5] Madhwani T., McBain A.J. (2011). Compositional modification of nascent *in vitro* dental plaques by human host-defence peptides. FEMS Immunol. Med. Microbiol.

[6] Yao Y., Berg E.A., Costello C.E., Troxler R.F., Oppenheim F.G. (2003). Identification of protein components in human acquired enamel pellicle and whole saliva using novel proteomics approaches. J. Biol. Chem.

[7] Hannig C., Hannig M., Attin T. (2005). Enzymes in the acquired enamel pellicle. Eur. J. Oral Sci.

[8] Lee J.-Y., Chung J.-W., Kim Y.-K., Chung S.-C., Kho H.-S. (2007). Comparison of the composition of oral mucosal residual saliva with whole saliva. Oral Dis.

[9] Vitorino R., Calheiros-Lobo M.J., Duarte J.A., Domingues P.M., Amado F.M.L. (2008). Peptide profile of human acquired enamel pellicle using MALDI tandem MS. J. Sep. Sci.

[10] McDonald E.E., Goldberg H.A., Tabbara N., Mendes F.M., Siqueira W.L. (2011). Histatin 1 resists proteolytic degradation when adsorbed to hydroxyapatite. J. Dent. Res.

[11] Soares R.V., Lin T., Siqueira C.C., Bruno L.S., Li X., Oppenheim F.G., Offner G., Troxler R.F. (2004). Salivary micelles: Identification of complexes containing MG2, sIgA, lactoferrin, amylase, glycosylated proline-rich protein and lysozyme. Arch. Oral Biol.

[12] Ogawa Y., Miura Y., Harazono A., Kanai-Azuma M., Akimoto Y., Kawakami H., Yamaguchi T., Toda T., Endo T, Tsubuki M. (2011). Proteomic analysis of two types of exosomes in human whole saliva. Biol. Pharm. Bull..

[13] Gorr S.-U. (2009). Antimicrobial peptides of the oral cavity. Periodontology 2000.

[14] Csermely P., Ágoston V., Pongor S. (2005). The efficiency of multi-target drugs: The network approach might help drug design. Trends Pharmacol. Sci.

[15] Vylkova S., Li X.S., Berner J.C., Edgerton M. (2006). Distinct antifungal mechanisms: β-defensins require *Candida albicans* Ssa1 protein, while Trk1p mediates activity of cystein-free cationic peptides. Antimicrob. Agents Chemoter.

[16] Leito T.D., Ligtenberg A.J.M., Nazmi K., Veerman E.C.I. (2009). Identification of salivary components that induce transition of hyphae to yeast in *Candida albicans*. FEMS Yeast Res.

[17] White M.R., Helmerhorst E.J., Ligtenberg A., Karpel M., Tecle T., Siqueira W.L., Oppenheim F.G., Hartshorn K.L. (2009). Multiple components contribute to ability of saliva to inhibit influenza viruses. Oral Microbiol. Immunol.

[18] Brandtzaeg P. (2007). Do salivary antibodies reliably reflect both mucosal and systemic immunity?. Ann. NY Acad. Sci.

[19] Bandtzaeg P., Korsund F.R. (1984). Significance of different J chain profiles in human tissues: Generation of IgA and IgM with binding site for secretory component is related to the J chain expressing capacity of the total local immunocyte population, including IgG and IgD producing cells, and depends on the clinical state of the tissue. Clin. Exp. Immunol.

[20] Kinane D.F., Lappin D.F., Koulouri O., Buckley A. (1999). Humoral immune responses in periodontal disease may have mucosal and systemic immune features. Clin. Exp. Immunol.

[21] Mestecky J., McGhee J.R., Arnold R.R. (1978). Selective induction of an immune response in human external secretions by ingestion of bacterial antigen. J. Clin. Invest.

[22] Proctor G.B., Carpenter G.H., Segawa A., Garrett J.R., Ebersole L. (2003). Constitutive secretion of immunoglobulin A and other proteins into lumina of unstimulated submandibular glands in anaesthetized rats. Exp. Physiol.

[23] Carpenter G.H., Proctor G.B. (2000). Double electrophoretic separation and lectin analyses of the component chains of secretory immunoglobulin A from human saliva. Electrophoresis.

[24] Johansen F.-E., Brandtzaeg P. (2004). Transcriptional regulation of the mucosal IgA system. Trends Immunol.

[25] Phalipon A., Cortésy B. (2003). Novel functions of the polymeric Ig receptor: Well beyond transport of immunoglobulins. Trends Immunol.

[26] De Oliveira I.R., de Araújo A.N., Bao S.N., Giugliano L.G. (2001). Binding of lactoferrin and free secretory component to enterotoxigenic *Escherichia coli*. FEBS Microbiol. Lett.

[27] Dallas S.D., Rolfe R.D. (1998). Binding of *Clostridium difficile* toxin A to human milk secretory component. J. Med. Microbiol.

[28] Phalipon A., Cardona A., Kraehenbuhl J.-P., Edelman L., Sansonetti P.J., Corthésy B. (2002). Secretory component: A new role in secretory IgA-mediated immune exclusion *in vivo*. Immunity.

[29] Brandtzaeg P., Fjellanger I., Gjeruldsen S.T. (1968). Adsorption of immunoglobulin A onto oral bacteria *in vivo*. J. Bacteriol.

[30] Shugars D.C., Wahl S.M. (1998). The role of the oral environment in HIV-1 transmission. J. Am. Dent. Assoc.

[31] Carrero J.C., Cervantes-Rebolledo C., Aguilar-Diaz H., Diaz-Gallardo M.Y., Laclette J.P., Morales-Montor J. (2007). The role of secretory immune response in the infection by *Entamoeba histolytica*. Parasite Immunol.

[32] Brandtzaeg P. (2007). Induction of secretory immunity and memory at mucosal surfaces. Vaccine.

[33] Wines B.D., Hogarth P.M. (2006). IgA receptors in health and disease. Tissue Antigens.

[34] Babior B.M., Takeuchi C., Ruedi J., Gutierrez A., Wentworth P. (2003). Investigating antibody-catalyzed ozone generation by human neutrophils. Proc. Natl. Acad. Sci. USA.

[35] Wentworth P., Jones L.H., Wentworth A.D., Zhu X., Larsen N.A., Wilson I.A., Xu X., Goddard W.A., Janda K., Eschenmoser A. (2001). Antibody catalysis of the oxidation of water. Science.

[36] Wentworth P., McDunn J.E., Wentwoth A.D., Takeuchi C., Nieva J., Jones T., Bautista C., Ruedi J.M., Gutierrez A., Janda K.D. (2002). Evidence for antibody-catalyzed ozone formation in bacterial killing and inflammation. Science.

[37] Nieva J., Wentworth P. (2004). The antibody-catalyzed water oxidation pathway—A new chemical arm to immune defense?. Trends Biochem. Sci..

[38] Nathan C. (2002). Catalytic antibody bridges innate and adaptive immunity. Science.

[39] Nieva J., Kerwin L., Wentworth A.D., Lerner R.A., Wentworth P. (2006). Immunoglobulins can utilize riboflavin (Vitamin B2) to activate the antibody-catalyzed water oxidation pathway. Immunol. Lett..

[40] Yamashita K., Miyoshi T., Arai T., Endo N., Itoh H., Makino K., Mizugishi K., Uchiyama T., Sasada M. (2008). Ozone production by amino acids contributes to killing of bacteria. Proc. Natl. Acad. Sci. USA.

[41] Fernandez M.I., Pedron T., Tournebize R., Olivo-Marin J.-C., Sansonetti P.J., Phalipon A. (2003). Anti-inflammatory role for intracellular dimeric immunoglobulin A by neutralization of lipopolysaccharide in epithelial cells. Immunity.

[42] Asea A. (2005). Stress proteins and initiation of immune response: Chaperokine activity of Hsp70. Exerc. Immunol. Rev.

[43] Fábián T.K., Gáspár J., Fejérdy P., Kaán B., Bálint M., Csermely P., Fejérdy P (2003). Hsp70 is present in human saliva. Med. Sci. Monitor.

[44] Fábián T.K., Tóth Z., Fejérdy L., Kaán B., Csermely P., Fejérdy P. (2004). Photo-acoustic stimulation increases the amount of 70 kDa heat shock protein (Hsp70) in human whole saliva. A pilot study. Int. J. Psychophysiol.

[45] Calderwood S.K., Mambula S.S., Gray P.J., Theriault J.R. (2007). Extracellular heat shock proteins in cell signaling. (Minireview). FEBS Lett..

[46] Fábián T.K, Sőti C., Nguyen M.T., Csermely P., Fejérdy P., Morel E., Vincent C. (2008). Expected functions of salivary HSP70 in the oral cavity. Heat Shock Proteins: New Research.

[47] Anand P.K., Anand E., Bleck C.K.E., Anes E., Griffiths G (2010). Exosomal Hsp70 induces a pro-inflammatory response to foreign particles including mycobacteria. PLoS One.

[48] Nguyen M.T., Fábián T.K., Singh M., Csermely P., Sőti C. (2008). Bacterial binding and opsonizing effect of extracellular Hsp70. (Abstract No: YSF-85). FEBS J.

[49] Campisi J., Leem T.H., Fleshner M. (2003). Stress-induced extracellular Hsp72 is a functionally significant danger signal to the immune system. Cell Stress Chaperones.

[50] Multhoff G. (2002). Activation of natural killer cells by heat shock protein 70. Int. J. Hyperth.

[51] Prohászka Z., Sing M., Nagy K., Kiss E., Lakos G., Duba J., Füst G. (2002). Heat shock protein 70 is a potent activator of the human complement system. Cell Stress Chaperones.

[52] Srivastava P.K. (2002). Heat shock proteins in innate and adaptive immunity. Nat. Rev. Immunol.

[53] Cutler C.W., Jotwani R. (2006). Dendritic cells at the oral mucosal interface. J. Dent. Res.

[54] Ito H., Takekoshi T., Miyauchi M., Ogawa I., Takata T., Nikai H., Takemoto K. (1998). Three-dimensional appearance of Langerhans cells in human gingival epithelium as revealed by confocal laser scanning microscopy. Arch. Oral Biol.

[55] Johnson A.D., Tytell M. (1993). Exogenous Hsp70 becomes cell associated, but not internalized by stressed arterial smooth muscle cells. In Vitro Cell. Dev. Biol. Anim.

[56] Guzhova I., Kislyakova K., Moskaliova O., Fridlanskaya I., Tytell M., Cheetham M., Margulis B. (2001). *In vitro* studies show that, Hsp70 can be released by glia and that exogenous Hsp70 can enhance neuronal stress tolerance. Brain Res.

[57] Boulanger J., Faulds D., Eddy E.M., Lingwood C.A. (1995). Members of the 70 kDa heat shock protein family specifically recognize sulfoglycolipids: Role in gameterecognition and mycoplasma-related infertility. J. Cell. Physiol.

[58] Guzhova I.V., Arnoldt A.C., Darieva Z.A., Kinev A.V., Lauskaia E.B., Nilsson K., Bozhkov V.M., Voronin A.P., Margulis B.A. (1998). Effects of exogenous stress protein 70 on the functional properties of human promonocytes through binding to cell surface and internalization. Cell Stress Chaperones.

[59] Asea A., Kraeft S.K., Kurt-Jones E.A., Stevenson M.A., Chen L.B., Finberg R.W., Koo G.C., Calderwood S.K. (2000). Hsp70 stimulates cytokine production through a CD14-dependent pathway, demonstrating its dual role as a chaperone and cytokine. Nat. Med.

[60] Asea A., Rehli M., Kabingu E., Boch J.A., Bare O., Auron P.E., Stevenson M.A., Calderwood S.K. (2002). Novel signal transduction pathway utilized by extracellular HSP70: Role of toll like receptor (TLR) 2 and TLR4. J. Biol. Chem.

[61] Li X.S., Reddy M.S., Baev D., Edgerton M. (2003). Candida albicans Ssa1/2p is the cell envelope binding protein for human salivary histatin 5. J. Biol. Chem.

[62] Li X.S., Sun J.N., Okamoto-Shibayama K., Edgerton M. (2006). Candida albicans cell wall Ssa proteins bind and facilitate import of salivary Histatin 5 required for toxicity. J. Biol. Chem.

[63] Wiesner J., Vilcinskas A. (2010). Antimicrobial peptides. The ancient arm of the human immune system. Virulence.

[64] Diamond D.L., Kimball J.R., Krisanaprakornkit S., Ganz T., Dale B.A. (2011). Detection of beta-defensins secreted by human oral epithelial cells. J. Immunol. Methods.

[65] Brodgen K.A. (2005). Antimicrobial peptides: Pore formers or metabolic inhibitors in bacteria?. Nat. Rev. Microbiol.

[66] Jenssen H., Hamill P., Hancock R.E. (2006). Peptide antimicrobial agents. Clin. Microbiol. Rev.

[67] Johnson D.A., Yeh C.K., Dodds M.W.J. (2000). Effect of donor age on the concentration of histatins in human parotid and submandibular/sublingual saliva. Arch. Oral Biol.

[68] Oudhoff M.J., Bolscher J.G., Nazmi K., Kalay H., van’t Hof W., Nieuw Amerongen A.V., Veerman E.C.I. (2008). Histatins are the major wound-closure stimulating factors in human saliva as identified in cell culture assay. FASEB J.

[69] Grogan J., McKnight C.J., Troxler R.F., Oppenheim F.G. (2001). Zinc and copper bind to unique sites of histatin 5. FEBS Lett.

[70] Gusman H., Travis J., Helmerhorst E.J., Potempa J., Troxler R.F., Oppenheim F.G. (2001). Salivary histatin 5 is an inhibitor of both host and bacterial enzymes implicated in periodontal disease. Infect. Immun.

[71] Nishikata M., Kanehira T., Oh H., Tani H., Tazaki M., Kuboki Y. (1991). Salivary hystatin as an inhibitor of protease produced by the oral bacterium *Bacteroides gingivalis*. Biochem. Biophys. Res. Comm.

[72] Shimotoyodome A., Kobayashi H., Tokimitsu I., Matsukobo T. (2006). Statherin and histatin 1 reduce parotid saliva promoted Streptococcus mutans strain MT8148 adhesion to hydroxyapatite surfaces. Caries. Res.

[73] Bennick A. (2002). Interaction of plant polyphenols with salivary proteins. Crit. Rev. Oral Biol. Med.

[74] Van der Kraan M.I., Groenink J., Nazmi K., Veerman E.C., Bolscher J.G., Nieuw Amerongen A.V. (2004). Lactoferrampin: A novel antimicrobial peptide in the *N*-1-domain of bovine lactoferrin. Peptides.

[75] Bolscher J.G., Adao R., Nazmi K., van der Keybus P.A., van’t Hof W., Nieuw Amerongen A.V., Bastos M., Veerman E.C.I. (2009). Bactericidal activity of LFchimera is stronger and less sensitive to ionic strength than its constituent lactoferricin and lactoferrampin peptides. Biochimie.

[76] Välimaa H., Tenovuo J., Waris M., Hukkanen V. (2009). Human lactoferrin but not lysozyme neutralizes HSV-I and inhibits HSV-I replication and cell-to-cell spread. Virol. J.

[77] Boman H.G. (2003). Antibacterial peptides: Basic facts and emerging concepts. J. Intern. Med.

[78] Heilborn J.D., Nilsson M.F., Kratz G., Weber G., Sorensen O., Borregaard N., Stale-Backdahl M. (2003). The cathelicidin anti-microbial peptide LL-37 is involved in re-epithelialization of human skin wounds and is lacking in chronic ulcer epithelium. J. Invest. Dermatol.

[79] Moreau T., Baranger K., Dadé S., Dallet-Choisy S., Guyot N., Zani M.L. (2008). Multifaceted roles of human elafin and secretory leukocyte proteinase inhibitor (SLPI), two serine protease inhibitors of the chelonianin family. Biochimie.

[80] Jana N.K., Gray L.R., Shugars D.C. (2005). Human immunodeficiency virus type 1 stimulates the expression and production of secretory leukocyte protease inhibitor (SLPI) in oral epithelial cells: A role of SLPI in innate mucosal immunity. J. Virol.

[81] Williams S.E., Brown T.I., Roghanian A., Sallenave J.-M. (2006). SLPI and elafin: One glove, many fingers. Clin. Sci.

[82] Gröschl M., Wendler O., Topf H.-G., Bohlender J., Köhler H. (2009). Significance of salivary adrenomedullin in the maintenance of oral health: Stimulation of oral cell proliferation and antibacterial properties. Regul. Peptides.

[83] Kapas S., Bansal A., Bhargava V., Maher R., Malli D., Hagi-Pavli E., Allaker R.P. (2001). Adrenomedullin expression in pathogen-challenged oral epithelial cells. Peptides.

[84] Allaker R.P., Kapas S. (2003). Adrenomedullin and mucosal defense: Interaction between host and microorganism. Regul. Peptides.

[85] Allaker R.P., Grosvenor P.W., McAnerney D.C., Sheehan B.E., Srikanta B.H., Pell K., Kapas S. (2006). Mechanisms of adrenomedullin antimicrobial action. Peptides.

[86] Veerman E.C., van den Keybus P.A., Vissink A., Nieuw Amerongen A.V. (1996). Human glandular salivas: Their separate collection and analysis. Eur. J. Oral Sci.

[87] Laible N.J., Germaine G.R. (1985). Bactericidal activity of human lysozyme, muramidase-inactive lysozyme and cationic polypeptides against Streptococcus sanguis and Streptococcus faecalis: Inhibition by chitin oligosaccharides. Infect. Immun.

[88] Ibrahim H.R., Thomas U., Pellegrini A. (2001). A helix-loop-helix peptide at the upper lip of the active site cleft of lysozyme confers potent antimicrobial activity with membrane permabilization action. J. Biol. Chem.

[89] Marquis G., Garzon S., Strykowski H., Auger P. (1991). Cell walls of normal and lysozyme-damaged blastoconidia of *Candida albicans*: Localization of surface factor 4 antigen and vicinal-glycol staining. Infect. Immun.

[90] Lee-Huang S., Huang P.L., Sun Y., Huang P.L., Kung H.-F., Blithe D.L., Chen H.-C. (1999). Lysozyme and RNases as anti-HIV components in β-core preparations of human chorionic gonadotropin. Proc. Natl. Acad. Sci. USA.

[91] Sava G., Benetti A., Ceschia V., Pacor S. (1989). Lysozyme and cancer: Role of exogenous lysozyme as anticancer agent (review). Anticancer Res.

[92] Choi S., Baik J.E., Jeon J.H., Cho K., Seo D.-G., Kum K.-Y., Yun C.-H., Han S.H. (2011). Identification of Porphiromonas gingivalis lipopolysaccharide-binding proteins in human saliva. Mol. Immunol.

[93] Gasior-Chrzan B., Falk E.S. (1992). Lysozyme and IgA concentrations in serum and saliva from psoriatic patients. Acta Derm. Venereol. (Stockh.).

[94] Geetha C., Venkatesh S.G., Dunn B.H., Gorr S.U. (2003). Expression of anti-bacterial activity of human parotid secretory protein (PSP). Biochem. Soc. Trans.

[95] Shiba H., Venkatesh S.G., Gorr S.U., Barbieri G., Kurihara H., Kinane D.F. (2005). Parotid secretory protein is expressed and inducible in human gingival keratinocytes. J. Periodontal Res.

[96] Vargas P.A., Speight P.M., Bingle C.D., Barrett A.W., Bingle L. (2008). Expression of PLUNC family members in benign and malignant salivary gland tumors. Oral Dis.

[97] Bosch J.A., Veerman E.C.I., de Geus E.J., Proctor G.B. (2011). α-Amylase as a reliable and convenient measure of sympathetic activity: Don’t start salivating just yet!. Psychoneuroendocrinology.

[98] Scannapieco F.A., Torres G., Levine M.J. (1993). Salivary α-amylase: Role in dental plaque and caries formation. Crit. Rev. Oral Biol. Med.

[99] Vitorino R., Lobo M.J.C., Ferrer-Correira A., Dubin J.R., Tomer K.B., Domingues P.M., Amado M.L. (2004). Identification of human whole saliva protein components using proteomics. Proteomics.

[100] Scannapieco F.A., Torres G., Levine M.J. (1995). Salivary amylase promotes adhesion of oral streptococci to hydroxyapatite. J. Dent. Res.

[101] Rogers J.D., Palmer R.J., Kolenbrander P.E., Scannapieco F.A. (2001). Role of Streptococcus gordonii amylase-binding protein A in adhesion to hydroxyapatite, starch metabolism, and biofilm formation. Infect. Immun.

[102] Okahashi N., Nakata M., Terao Y., Isoda R., Sakurai A., Sumitomo T., Yamaguchi M., Kimura R.K., Oiki E., Kawabata S., Ooshima T. (2011). Pili of oral Streptococcus sanguinis bind to salivary amylase and promote the biofilm formation. Microb. Pathog.

[103] Mellersh A., Clark A., Hafiz S. (1979). Inhibition of Neisseria gonorrhoeae by normal human saliva. Br. J. Ven. Dis.

[104] Sato K., Tokuhisa S., Inaba Y. (1995). Effect of enzymes on the growth of human and animal rotaviruses. J. Vet. Med. Sci.

[105] Dickinson D.P. (2002). Salivary (SD-type) cystatins: Over one billion years in making—But to what purpose?. Crit. Rev. Oral Biol. Med.

[106] Blankenvoorde M.F., Henskens Y.M., van der Weijden G.A., van den Keijbus P.A., Veerman E.C., Nieuw Amerongen A.V. (1997). Cystatin A in gingival crevicular fluid of periodontal patients. J. Periodontal Res.

[107] Blankenvoorde M.F., van’t Hof W., Walgreen-Weterings E., vanSteenbergen T.J., Brand H.S., Veerman E.C., Nieuw-Amerongen A.V. (1998). Cystatin and cystatin-derived peptides have antibacterial activity against the pathogen Porphiromonas gingivalis. Biol. Chem.

[108] Schwartz S.S., Zhu W.X., Sreebny L.M. (1995). Sodium dodecyl sulphate-polyacrylamide gel electrophoresis of human whole saliva. Arch. Oral Biol.

[109] Tenovuo J. (2002). Antimicrobial agents in saliva. Protection for the whole body. J. Dent. Res.

[110] Chang W.-I., Chang J.-Y., Kim Y.-Y., Lee G., Kho H.S. (2011). MUC1 expression in the oral mucosal epithelial cells of the elderly. Arch. Oral Biol.

[111] Denny P.C., Denny P.A., Klauser D.K., Hong S.H., Navazesh M., Tabak L.A. (1991). Age related changes in mucins from hguman whole saliva. J. Dent. Res.

[112] Veerman E.C.I., Valentijl-Benz M., van den Keybus P.A.M., Rathman W.M., Sheehan J.K., Nieuw Amerongen A.V. (1991). Immunochemical analysis of high molecular-weight human salivary mucins (MG1) using monoclonal antibodies. Arch. Oral Biol.

[113] Pramanik R., Osalian S.M., Challacombe S.J., Urquhart D., Proctor G.B. (2010). Protein and mucin retention on oral mucosal surfaces in dry mouth patients. Eur. J. Oral Sci.

[114] Lis M., Liu T.T., Barker K.S., Rogers P.D., Bobek L.A. (2010). Antimicrobial peptide MUC7 12-mer activates the calcium/calcineurin pathways of Candida albicans. FEMS Yeast Res.

[115] Ihalin R., Loimaranta V., Tenovuo J. (2006). Origin, structure, and biological activities of peroxidases in human saliva. Arch. Biochem. Biophys.

[116] Welk A., Meller CH., Schubert R., Schwahn C., Kramer A., Below H (2009). Effect of lactoperoxidase on the antimicrobial effectiveness of the thiocyanate hydrogen peroxide combination in a quantitative suspension test. BMC Microbiol.

[117] Ashby M.T. (2008). Inorganic chemistry of defensive peroxidases in the human oral cavity. J. Dent. Res.

[118] Geiszt M., Witta J., Baffi J., Lekstrom K., Leto T.L. (2003). Dual oxidases represent novel hydrogen peroxide sources supporting mucosal surface host defense. FASEB J.

[119] Rada B., Leto T.L. (2008). Oxidative innate immune defenses by Nox/Duox family NADPH oxidases. Contrib. Microbiol.

[120] Jensen J.L., Lamkin M.S., Troxler R.F., Oppenheim F.G. (1991). Multiple forms of atatherin in human salivary secretions. Arch. Oral Biol.

[121] Schwartz S.S., Hay D.I., Schluckebier S.K. (1992). Inhibitio of calcium phosphate precipitation by human salivary statherin: Structure-activity relationships. Calcif. Tissue Int.

[122] Proctor G.B., Hamdan S., Carpenter G.H., Wilde P. (2005). A statherin and calcium enriched layer at the air interface of human parotod saliva. Biochem. J.

[123] Ligtenberg A.J., Veerman E.C., Nieuw Amerongen A.V., Mollenhauer J. (2007). Salivary agglutinin/glycoprotein-340/DMBT1: A single molecule with variable composition and with different functions in infection, inflammation and cancer. Biol. Chem.

[124] Hartshorn K.L., Ligtenberg A., White M.R., van Eijk M., Hartshorn M., Pemperton L., Holmskov U., Crouch E. (2006). Salivary agglutinin and lung scavenger receptor cysteine-rich glycoprotein 340 have broad anti-influenza activities and interactions with surfactant protein D that vary according to donor source and sialylation. Biochem. J.

[125] Edwards A.M., Manetti A.G.O., Falugi F., Zingaretti C., Capo S., Buccato S., Bensi G., Telford J.L., Margarit I., Grandi G. (2008). Scavenger receptor gp340 aggregates group A streptococci by binding pili. Mol. Microbiol.

[126] Malamud D., Abrams W.R., Barber C.A., Weissman D., Rehtanz M., Golub E. (2011). Antiviral activities in human saliva. Adv. Dent. Res.

[127] Corbin B.D., Seeley E.H., Raab A., Feldmann J., Miller M.R., Torres V.J., Anderson K.L., Dattilo B.M., Dunman P.M., Gerads R. (2008). Metal chelation and inhibition of bacterial growth in tissue abscesses. Science.

[128] Kido J., Nakamura T., Kido R., Ohishi K., Yamauchi N., Kataoka M., Nagata T. (1999). Calprotectin in gingival crevicular fluid correlated with clinical and biochemical markers of periodontal disease. J. Clin. Periodontol.

